# What Do People Know about Food, Nutrition and Health?—General Nutrition Knowledge in the Austrian Population

**DOI:** 10.3390/nu14224729

**Published:** 2022-11-09

**Authors:** Marlies Gruber, Chinyere Gina Iwuchukwu, Elisabeth Sperr, Jürgen König

**Affiliations:** 1 Forum. Ernährung Heute, Verein zur Förderung von Ernährungsinformationen Schwarzenbergplatz 6, 1037 Vienna, Austria; 2 Department of Nutritional Sciences, University of Vienna, Josef-Holaubek-Platz 2, 1090 Vienna, Austria

**Keywords:** nutrition, food, knowledge, literacy, questionnaire, diet, Austria

## Abstract

Background: Only limited information is available on the nutrition knowledge of the general Austrian population and how this relates to the nutrition knowledge of health professionals (medical doctors, pharmacists, nutritionist, dieticians) and school teachers. Methods: Adolescents and adults at the age of 14–75 years (*n* = 1000), medical doctors (*n* = 307), pharmacists (*n* = 295), nutritionists (*n* = 124), dieticians (*n* = 160) and school teachers (*n* = 873) completed an online survey using a German version of the General Nutrition Knowledge Questionnaire-Revised (GNKQ-R) including self-reported data on sex, age, BMI, and health status. Adolescent and adult participants were recruited by a research agency to be representative for the Austrian population for age, sex, and education. A convenience sample was used for health professionals (medical doctors, pharmacists, nutritionist, dieticians) and school teachers. All participants completed a computer-assisted web-based interviewing (CAWI) survey. Results: Total scores for nutrition knowledge of the general population (61.4%) were significantly lower than scores from all other groups (medical doctors 81.3%, pharmacists 83.0%, dieticians 87.5%, nutritionists 85.6%, school teachers 74.5%). The main drivers for better nutritional knowledge were female sex, higher age, and higher level of education, while BMI classification and self-reported health status had no impact. In regard to single questions, the most striking result was the misclassification of sugar as the nutrient with the most calories by 41.4% of the general population while only 29.0% correctly identified fat to be the nutrient with the most calories. Conclusions: The nutritional knowledge of the general population should be significantly improved in order to lay a basis for better dietary behavior. In view of the relatively low scores of teachers, their nutrition education should be improved in order to enable transfer of sound education in schools.

## 1. Introduction

Over the last years, the burden of non-communicable diseases such as obesity, diabetes, cancer and cardiovascular diseases increased rapidly worldwide [1]. In Austria, 41.0% of the population are overweight respectively obese according to the latest nutrition report from the year 2017 [2]. Overweight and obesity occur more often in older age groups than in younger ones. For example, every fourth man aged 15 to under 25 is overweight, but between the ages of 51 and 65 it is already every second man. The relationship between weight and age is present in both sexes. In 2020 the two most common causes of death in Austria were cardiovascular diseases (accounting for 35.7% of all deaths) and cancer (accounting for 22.9%). Together they were responsible for almost six out of ten deaths in the country [3].

Diet and lifestyle are known to play a significant role in the prevention of these nutrition-related diseases [1]. Hence, a plan of actions to address and change unhealthy dietary patterns is needed. There are many determinants of food choices, ranging for example from personal preferences to culture or economic status [4,5]. Nevertheless, the basis for healthy food habits is nutrition knowledge. It has the potential to promote diversified dietary patterns [6,7], thus to enhance nutrition status and to reduce the risk of developing nutrition-related diseases in the long term. On the other hand, a lack of knowledge and awareness for the importance of a balanced diet may contribute to poor diet practices [8]. Furthermore, nutrition knowledge leads to better understanding of dietary guidelines and enables consumers to take action in their daily lives [9]. Nutrition knowledge is also known to be influenced by a varied range of factors including sex, age, level of education and socio-economic status [8]. Previous research has reported that men have a lower level of nutrition knowledge than women, which may be related to women having a stronger interest in nutrition [8,10]. Moreover, there is a positive relationship between nutrition knowledge and education [8]. This comes along with other positive health behaviours such as lower prevalence of smoking or increased physical activity [11,12,13]. In addition to these factors, cultural differences, history of nutrition and public health policies, but also retailing initiatives in positioning products based on their nutritional properties were suggested to affect how people acquire knowledge about food and health [14,15]. Despite the fundamental role of nutrition knowledge and the ongoing debate about nutrition education in schools, no internationally comparable evaluation of the current status was performed in any of the German-speaking countries, namely Austria, Germany or Switzerland, till date. Thus, the aim of this study was to collect valid and internationally comparable data on the current status of nutrition knowledge in the general Austrian population and to compare the level of nutrition knowledge with that of health professionals (medical doctors, pharmacists, dieticians, nutritionists) and school teachers. The latter groups do play a crucial role in transferring knowledge, additionally they act as important role models. Therefore, their skills are pivotal for successful food and nutrition literacy, even if the subjects they teach into do not cover these topics in detail.

## 2. Materials and Methods

### 2.1. Participants

A total sample of 1000 individuals (502 male, 498 female) was approached through the “Online Access Panel” of the Austrian research agency “Marketagent” (Marketagent.com online research GmbH, Baden, Austria) and completed a computer-assisted web-based interviewing (CAWI) survey. The eligibility criteria included adolescents and adults aged 14–75 years who were living in Austria. The sample was representative for the overall Austrian population regarding sex, socioeconomic status and education. Data were collected during April 2021.

To assess nutrition knowledge of health professionals and school teachers, a convenience sample was recruited with support of their official professional associations, and by Social Media and personal networks of the authors. The sample comprises 886 health professionals (307 medical doctors, 295 pharmacists, 160 dieticians, 124 nutritionists) and 873 school teachers. These data were collected during July–September 2021 also using CAWI. The distribution of the respondents over the six groups, and across sex, age group, BMI group, and subjective health status are provided in Table 1.

### 2.2. Questionnaire

The General Nutrition Knowledge Questionnaire was developed in the 1990s by Parmenter and Wardle [16], validated for the UK population and has also been adapted and validated for other populations [17]. It was revised by Kliemann et al. in 2016 [18] and this revised version has also been previously validated and used to measure nutrition knowledge in different study populations [19,20,21,22,23,24]. The General Nutrition Knowledge Questionnaire-Revised (GNKQ-R) used for this survey features four sections of nutrition related questions: (1) questions on knowledge about expert advice (9 questions, maximum score 18 points), (2) questions on food groups and nutrients (10 questions, maximum score 36 points), (3) questions on healthy food choices (13 questions, maximum score 13 points), and (4) questions on diet related health problems and weight management (16 questions, maximum score 21 points). The overall maximum score across all sections is 88 points. The final section (5) covers questions on sociodemographic items (gender, age, education, marital status, number of children) and health indicators (self-reported body weight and body height, rating of health status).

The questionnaire was translated from English into German and the accuracy of the translation was checked by back translation to English. Differences between the original English version and the back translated version were checked and adapted. Slight modifications were made in some questions (Section 3, questions 3 and 4, Supplementary Table S1) to adjust the rather typical British meals with similar dishes in respect to food composition to better meet typical Austrian eating habits. Before dissemination to both samples (general Austrian population, health professionals and school teachers) the questionnaire was tested in a separate sample on comprehensibility and clarity. The final German version of the questionnaire was then administered to the online participants by the research agency for the general Austrian population and to SoSci Survey (www.soscisurvey.de, Munich, Germany) for the sample of health professionals and school teachers. The 57 questions were presented in single-choice format. For each item, correct answers scored one point, incorrect responses none. The points were further summed to obtain the scores of all sections separately as well as the total score. Thus, a high score in a specific section or in total indicates a high nutrition knowledge. Demographic data including age, gender, education, height and weight were also collected. BMI was calculated on the self-reported data on body weight and height (BMI = body weight [kg]/(body height [m])^2^).

### 2.3. Data Analysis

All data were analysed using IBM SPSS Statistics 28.0.1.0 (IBM Corp., Armonk, NY, USA, 2021). One-way analysis of variance was used to compare means of the general population grouped by sex, BMI-group, age-group, and health status with Bonferroni post-hoc analysis. To adjust for the different distribution of sex and age in the group of health professionals and school teachers compared to the general population, an analysis of covariance was used to compare means of the total sample with sex and age as covariants and pairwise comparisons using Bonferroni-corrected post-hoc tests. The significance level for all test was α < 0.05.

## 3. Results

### 3.1. Nutrition Knowledge of the General Population

The overall score of the general population was 53.9 points out of 88 points (=61.4%) (Table 2) with the lowest score (20.7 out of 36 points, 57.4%) in Section 2 (food groups and nutrients), followed by Section 1 (nutritional recommendations, 11.1 out of 18 points, 61.1%), Section 3 (healthy food choices, 8.5 out of 13 points, 65.4%) and Section 4 (diet related health problems and weight management, 13.9 out of 21 points, 66.0%). 

**Figure 1:**
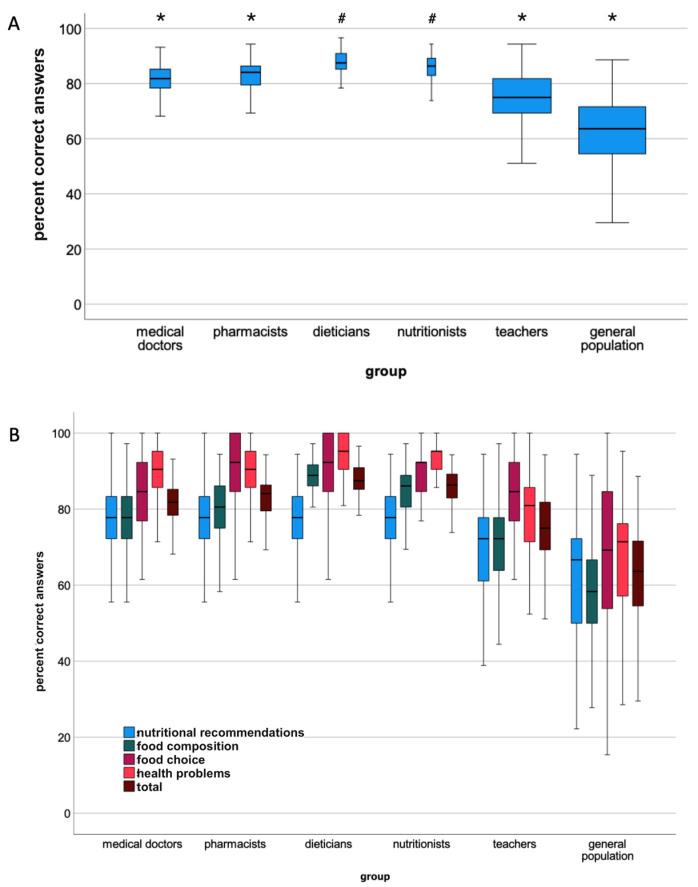
Box plots of scores (percent correct) in the study groups, line in box = median, lower and upper end of box = 25. and 75. percentile, whiskers minimum and maximum, (**A**): Box plot of total scores, with different box sizes indicating the sample size, all boxes labelled with equal symbols (*. #) are different from each other at *p* < 0.05, (**B**): Scores of all study groups for the four sections of the nutrition knowledge questionnaire (significant differences are provided in Table 2).

In all sections and across the entire questionnaire some individuals achieved scores lower than 20% (Figure 1A).

#### 3.1.1. Selected Results for Single Questions in the General Population

Only 59.8% of the general population responded correctly to the question, whether experts recommend that people should eat more of whole grains, while 31.8% believed that the same amount as now should be eaten (Supplemental Table S1). Between 70.0 and 88.9% were aware of the recommendations to eat less salty foods (78.1%), fatty foods (79.4%), processed red meat 70.0%), and food and drinks with added sugar (88.9%). Only 21.1% identified the recommendation to eat more than 5 servings of fruit and vegetables correctly, most people believed that 2 servings (31.2%) would be the correct answer. A clear difference for this question was found for male and female subjects: only 14.7% of male vs. 27.5% of female participants responded correctly. Many participants were not sure which type of fat should be consumed in higher or lower amounts (35.3% for trans fats, 26.8% for saturated fats and 26.6% for unsaturated fats, similar to the number of participants who were not sure what type of dairy foods should be consumed [20.2%]). Only 27.7% of the participants responded that experts say to drink reduced fat milk, while this knowledge was substantially better in people of higher age (χ^2^ (24) 37.9, *p* = 0.036, φ = 0.04).

About 36.9% of the participants believed that diet cola drinks and melons were high in added sugars, while 95.2% and 92.0% were correctly aware that ice cream and tomato ketchup are high in added sugars. In respect to salt content, 45.3% of participants considered bread to be low in salt, and 50.6% believed this to be also correct for breakfast cereals, with a relatively high number of participants (23.5%) not being sure, whether the salt content of breakfast cereals is high or low. The amount of dietary fibre was (falsely) estimated by 49.7% of individuals to be high in pasta, by 36.5% to be high in white rice and by 23.8% to be high in eggs. In contrast to dietary fibre, sugar and salt, the protein content of selected foods could be better associated with the correct foods, however, 28.3% considered butter to be a good source of protein while 57.4% considered baked beans not to be a good source of protein. A high level of uncertainty was also found for the classification of different fats: 41.2% believed that olive oil would be a polyunsaturated fat, while 29.5% believed this to be the case for sunflower oil. 48.6% of participants were aware that biscuits, cake and pastries are high in trans-fats, 32.0% however were not sure which of the foods (biscuits, cakes and pastries; fish; rapeseed oil, eggs) are high in trans-fats.

Sugar was the nutrient with the highest amount of calories for the same weight for 41.4% of the general population, whereas only 29.0% correctly identified fat as highest in calories and 15.4% were not sure, which of the nutrients sugar, fat, fibre, starch would be highest in calories. A higher rate of correct answers was observed in participants of higher age (58.6% at age 70–75) and a very low rate in young participants (19.5% at age 14–19 and 29.0% at age 20–29 years). Interestingly—and in contrast to the frequent wrong classification of sugar as highest in calories—many individuals (63.8%) were able to correctly identify sugar sources on the food label provided. Only 55.6% of individuals, however, were able to spot the correct information on the amount of kcal when a typical nutrition information label was presented.

The final section of the questionnaire was about health problems related to diet and weight management. Interestingly, the majority of individuals were able to correctly identify the relation between bowel disorders and low intake of fibre (66.4%), sugar and tooth decay (61.6%), salt and high blood pressure (48.9%), red meat and cancer (44.8%), trans fats and heart disease (69.3%), refined foods and diabetes (72.2%), animal fat and blood cholesterol (59.3%), high glycaemic index and white bread (60.3%). Nonetheless, between 8.9% (sugar and tooth decay) and 25.1% (high glycaemic index and white bread) were not sure about the correct answer. While 44.8% of all subjects considered eating less meat to be the expert recommendation to reduce the chances of getting cancer, a substantial number of individuals (37.3%) considered the avoidance of food additives to be the recommendation for cancer reduction. The majority of the general population (63.1%) agreed with the statement that people should eat a high protein diet to maintain a healthy weight and 51.7% (correctly) agreed with the statement that dietary fibre can decrease the chances of gaining weight.

Only 46.9% of the participants were able to classify a body mass index of 23 kg/m^2^ as normal, 35.6% were not sure and 10.7% classified this as overweight. 6% thought this body mass index indicates underweight. A body mass index of 31 kg/m^2^ was considered as overweight by 39.4%, as obese by 22.0%, and normal by 5.7%; 31.5% were not sure how to classify this body mass index. On the other hand, 80.1% of the participants were able to correctly identify the apple shape of the body to increase the risk of cardiovascular diseases.

#### 3.1.2. Factors of Influence on Nutrition Knowledge of the General Population

**Figure 2:**
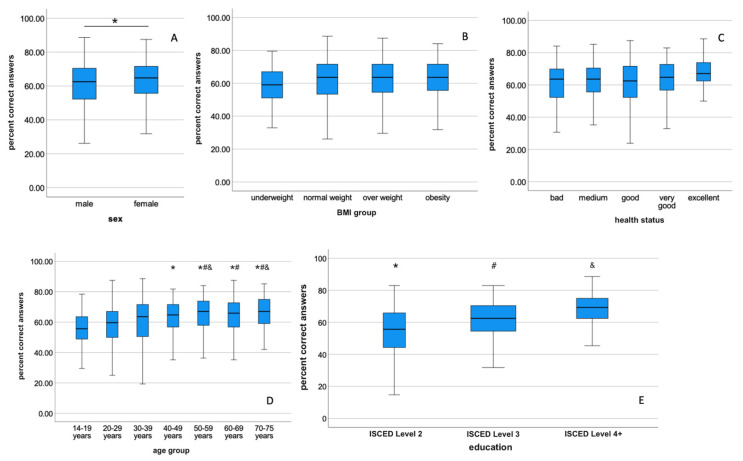
Box plots of total scores (percent correct) of the general population by sex (**A**), BMI group (**B**), self reported health status (**C**), age group (**D**), and level of education (**E**). (**A**): * indicates statistical difference between males and females at *p* < 0.05, (**D**): different symbols (*, #, &) indicate statistical differences by age group at *p* < 0.05, (**E**): different symbols (*, #, &) indicate statistical difference at *p* < 0.05.

Female participants achieved significantly better overall results than male participants (Figure 2A) and scored significantly better in Section 3 (healthy food choices) and 4 (diet related health problems and weight management, data not shown). Although female participants scored higher, the differences were relatively small (e.g., overall score 54.9 points vs. 53.0 points). No statistically significant differences were observed within the BMI groups (Figure 2B). Participants self-reporting better health scored better than those of less good health (Figure 2C), but these results were not statistically different. The overall scores were significantly higher with increasing age (Figure 2D), with the highest overall score achieved by participants in the age group 70–75 years. Generally, the age groups of 14–19 years and 20–29 years scored significantly lower than participants above 30 years. An increasing level of education (Figure 2E) also increased significantly the scores achieved. In total, the main drivers for better nutritional knowledge were female sex, higher age, and higher level of education.

### 3.2. Nutritional Knowledge of Health Professionals and School Teachers

Figure 1B shows the overall scores of health professionals (medical doctors, pharmacists, dieticians, nutritionists) and of school teachers compared to those of the general population. Dieticians (total score 87.5 ± 3.7%) and nutritionists (85.6 ± 6.3%) scored significantly better than all the other groups, followed by medical doctors (81.3 ± 5.7%) and pharmacists (82.9 ± 5.4%). While school teachers (74.5 ± 9.5%) scored significantly better than the general population (61.4 ± 14.1%), they also scored significantly lower than the groups of health professionals. The results from the four sections of the questionnaire are shown in Figure 1B for all groups. There were some differences between the groups for the four sections, although in all sections school teachers and the general population scored lowest, while dieticians and nutritionists scored highest (Table 2).

**Figure 3:**
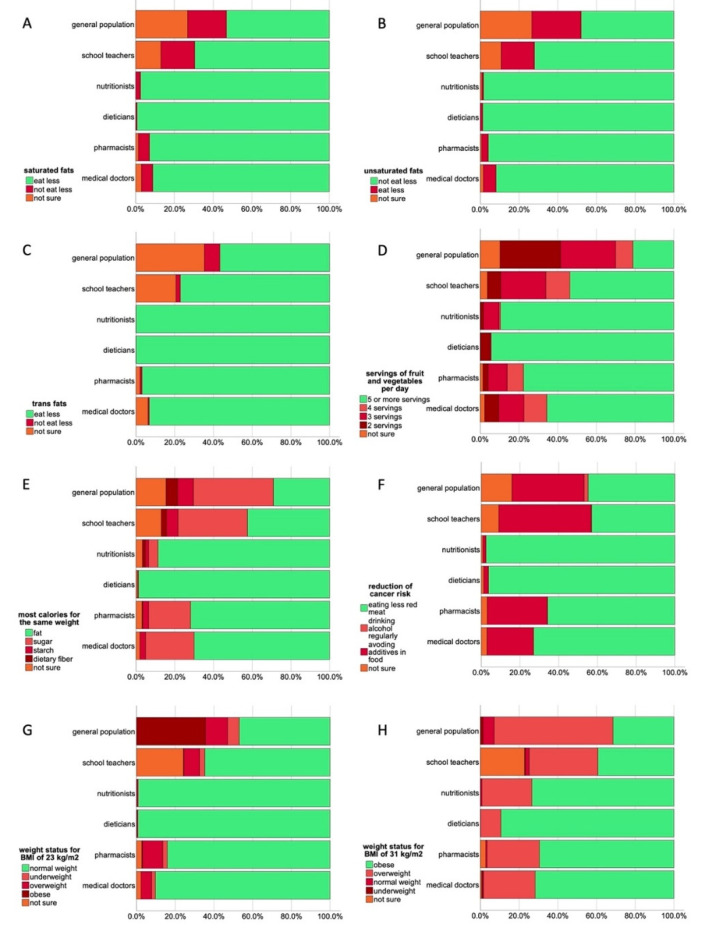
Responses (%) to selected questions by all study groups, green bars indicate correct answers, red shades wrong answers, orange bars “not sure”. (**A**), responses to question “Which of these types of fat do experts recommend to eat less of”–saturated fats (**B**), responses to question “Which of these types of fat do experts recommend to eat less of”–unsaturated fats (**C**), responses to question “Which of these types of fat do experts recommend to eat less of”–trans fats (**D**), responses to question “How many servings of fruit and vegetables per day do experts advise people to eat as a minimum?” (**E**), responses to question “Which one of the following nutrients has the most calories for the same weight of food”, (**F**), responses to question “Which of these options do experts recommend to reduce the chances of getting cancer”, (**G**), responses to question “If someone has a Body Mass Index (BMI) of 23 kg/m^2^, what would their weight status be?”, (**H**), responses to question “If someone has a Body Mass Index (BMI) of 31 kg/m^2^, what would their weight status be?”.

Figure 3 provides an overview of the responses of health professionals and school teachers to selected questions from the different sections of the questionnaire, again compared to the general population. Although the knowledge of these groups was better than the general population, there is still some degree of uncertainty even in well trained groups. Most health professionals were aware of the recommendation to eat less “saturated fats” (Figure 3A), not to reduce the consumption of “unsaturated fats” (Figure 3B), and to reduce the intake of “trans fats” (Figure 3C). School teachers had a higher degree of uncertainty about these recommendations or ticked the wrong answer. Similar as in the general population, although to a lower extent, a high number of health professionals considered a lower intake than 5 servings of fruit and vegetables as the expert recommendation (Figure 3D), even some nutritionists and dieticians were unaware of the correct answer. Again, school teachers showed the lowest number of correct answers.

That fat is highest in calories was best known by dieticians, while even 4.8% of nutritionists considered sugar to be the nutrient highest in calories. The number of wrong answers increased in pharmacists, medical doctors and school teachers, the latter reaching again a high number of incorrect answers (Figure 3E). Similar to the majority of the general population (63.1%) medical doctors (63.9%), pharmacists (67.1%) and school teachers (65.4%) agreed also with the statement that people should eat a high protein diet to maintain a healthy weight. Among the given options for the question on the recommendations to reduce the risk of developing cancer, both dieticians and nutritionists selected the reduced consumption of red meat as correct answer. This was also well known by medical doctors and pharmacists, but a substantial number from these professional groups decided that the avoidance of food additives would be the recommendation for cancer risk reduction. The avoidance of food additives was considered to be the correct recommendation by the majority of school teachers, which was an even higher number than those of the general population (Figure 3F).

The classification of a body mass index of 23 kg/m^2^ as normal weight (Figure 3G) was well known by dieticians and nutritionists, only 0.6 and 0.8%, respectively, were not able to respond correctly to this question. Increasing numbers of wrong answers were noted for medical doctors (9.8%), pharmacists (15.9%), and school teachers (35.3%). The participants were less able to correctly classify a body mass index of 31 kg/m^2^ as obese weight, even 10.6% of the dieticians and 25.8% of nutritionists considered this BMI still as overweight. Increasing numbers of incorrect answers were again found for pharmacists, medical doctors, and, to the highest extent, for school teachers (Figure 3H). Only 39.3% of school teachers were able to give the correct answer for this body mass index.

#### Health Status

In addition to the questions on nutrition knowledge, all participants were also asked to rate their present health status as poor, fair, good, very good or excellent. A poor health status was reported by 4.4% of the general population and a fair health status by 25.2%. Most participants (45.3%) considered their health status as good. Altogether, 70.4% stated a good, very good or excellent health status (45.3, 21.6, and 3.5%, resp.). However, the general population rated their health status significantly lower than health professionals and school teachers. It has to be noted, that dieticians and nutritionists were significantly younger and medical doctors, pharmacists and school teachers were significantly older than the general population.

## 4. Discussion

Food literacy has been defined by Vidgen and Gallegos in 2014 as “the scaffolding that empowers individuals, house-holds, communities or nations to protect diet quality through change and strengthen dietary resilience over time. It is composed of a collection of inter-related knowledge, skills and behaviours required to plan, manage, select, prepare and eat food to meet needs and determine intake” [25]. While this definition covers a broader range of different aspects of food literacy, knowledge about foods and their impact on our health is the part of the definition which is the most frequently used aspect in respect to food literacy according to a scoping review by Truman et al. [26]. Nutrition knowledge has been shown to be significantly associated with a “healthy diet” (e.g., fruit and vegetable consumption) [27]. In that study, medical doctors with better nutritional knowledge were 25 times more likely to consume adequate amounts of fruits and vegetables on a daily basis. Another study in Belgian women aged 18–39 years using the same instrument as in our study showed that better knowledge was related to better dietary behaviour [28]. It has to be noted, however, that sufficient nutrition knowledge alone may not be sufficient to modify eating behaviour [7,29].

Here, we present data on the nutritional knowledge of a representative sample of the general Austrian population as part of their food literacy and in comparison to the nutritional knowledge of health professionals and school teachers.

For a targeted approach to improve nutrition knowledge, it is important to identify possible gaps in the information perceived by the general public and to strengthen educational approaches based on these gaps. Our study does not provide a strategy for better educational approaches, but simply indicates a starting point. Our results show, that the general nutrition knowledge of the general population depends on gender, age, and level of education, which is in line with a range of similar studies [30]. Young, male and less educated persons scored worst in our study, whereas well educated, female participants of higher age scored best, which is not surprising considering that women typically have a higher interest in nutrition as being the person responsible for food purchasing and food preparation.

It is surprising that roughly three out of ten participants (29.0%) of the general population identify fat as the nutrient with the highest calorie content of all main nutrients and that many participants (41.4%) believe that sugar has got the most calories. This is most likely due to the ongoing discussion on the possible health impacts of high sugar consumption. It is therefore important to emphasize that mainly fat contributes to high energy intakes and that consumers should be aware of the importance of this nutrient for the energy balance. The recommendations for the intake of fruits and vegetables are just as little known (by every fifth participant). Lack of knowledge exists also when it comes to sources of salt and fiber and only every second person is aware of the association of salt intake and blood pressure as well as the potential of fiber in weight management. Similarly, the classification of the BMI into the weight categories of the WHO appears to be largely unknown to the general population, since only close to half of the participants were able to classify a BMI of 23 kg/m^2^ correctly as normal weight and only 22% could classify a BMI of 31 kg/m^2^ as obese. A very similar result on the inability to correctly identify this BMI as obese was found in the study by Balani et al. [31], who reported that only 28.1% of their participants were able to answer this question correctly. This clearly shows, that knowledge, which experts consider to be common, is of little relevance for the general population.

Health professionals, such as medical doctors and pharmacists are frequently the main source of information about nutrition related topics [32]. Therefore, it is vital that these professionals themselves have a profound knowledge of the information they transfer to their patients and clients. This applies even more to school teachers, who are supposed to set the basis for nutritional education in schools. Even though food and nutrition are not necessarily the subject they teach, their general nutritional knowledge influences the capacity for interdisciplinary information transfer. As expected, and as hoped, health professionals indeed had a better nutrition knowledge, which has also been shown by others [33]. Our data cannot answer how the gap between the knowledge of health professionals and that of the general population can be bridged. Ultimately, the only strategy is probably nutrition education starting as early as possible and being continued at all levels of the education system, including adult education. The rather low nutritional knowledge of teachers is worrying, as they are the most important multipliers in early nutrition education. The frequently suggested integration of nutritional education into school lessons can only be successful if the level of training of teachers in this area is significantly improved [34]. Overall, the results of our study give a clear and detailed picture of the nutrition knowledge in a large sample of the general Austrian population and the knowledge gaps compared to health professionals and school teachers.

### Strengths and Limitations

A major strength of our study is the data collected from a sample representative for the adult Austrian population in respect to age, gender, and socio-economic status. Another strength is the use of the validated revised version of the General Nutrition Knowledge Questionnaire, which has also been used in other studies and thus allows direct comparison.

It can be debated for a few questions, which answers are to be considered the correct ones (e.g., “Approximately how many alcoholic drinks is the maximum recommended per day”, correct answer 1 drink each for men and women; “Do health experts recommend that people should be eating more, the same amount, or less of the following foods”, correct answer fruit more). These questions, however, are only few and would not have had a major impact on the overall results if the correct answers were changed.

A limitation is the convenience sample of the other sub groups (medical doctors, pharmacists, nutritionists, dieticians, teachers). Although we had the support by the professional associations for those groups, the participation was voluntary and it can be assumed that individuals more interested in nutrition or more confident in their nutrition knowledge participated. It is likely, that lower scores would be achieved if a representative sample had been available. We also have no information on the specialization of the medical doctors—it is likely, that differences in nutrition knowledge are present between the medical fields. This is also the case for the subjects taught by school teachers. Nutrition related subjects are typically covered in the subjects biology, chemistry, or in dedicated subjects such as nutrition or home economics. We did not collect the information on the subject taught, again, it is likely, that there are differences in nutrition knowledge.

It has to be noted, that there is an age difference in the different professional groups due to the different duration of training and until employment. Typically, dieticians and nutritionists are younger than teachers, pharmacists and medical doctors. Since sex has also been found to be an influencing factor on nutrition knowledge and in particular the majority of nutritionists and dieticians typically classify themselves as females, this also may have had an impact on the results which also should be taken into account. While the percentages of females in the group of nutritionists (82.3% vs. 80.1% [35]), dieticians (97.5% vs. 90.0% [36]) and pharmacists (85.1% vs. 88.8% [37]) in our sample are similar to that in the population, the percentages of females in the group of teachers (84.2 vs. 71.7% [38]) and medical doctors are higher in our sample (73.9% vs. 48.5% [39]). We considered the bias caused by these factors by adjusting our statistical analysis to these covariants.

## 5. Conclusions

Nutritional knowledge based on the results of our study is generally in need of improvement, particularly in the general population and, due to their special role in nutrition education, also among school teachers. Even if nutritional knowledge as such is not the sole key to improving nutritional behaviour, greater attention should be paid to the fact that some details, which are obvious to experts, are much less known outside their fields. In medial communication about certain associations between nutrition and health, nutritional information should be handled more carefully, so that incorrect assessments, such as the energy content of fat and sugar, do not occur.

## Figures and Tables

**Table 1:** Sample characteristics (*n* = number of subjects, f = female, m = male).

Study Group	General Population			Health Professionals												School Teachers		
				Medical Doctors			Pharmacists			Dieticians			Nutritionists					
	total	m	f	total	m	f	total	m	f	total	m	f	total	m	f	total	m	f
*n*	1000	502	498	307	78	227	295	44	251	160	4	156	124	22	102	873	137	735
Age group																		
14–19	77	39	38	0	0	0	0	0	0	0	0	0	0	0	0	0	0	0
20–29	162	83	79	21	5	15	39	4	35	69	1	68	93	19	74	113	13	100
30–39	176	89	87	58	11	47	110	18	92	44	0	44	23	2	21	160	17	143
40–49	166	86	80	85	16	69	68	9	59	27	2	25	6	1	5	207	32	175
50–59	201	101	100	77	15	62	60	8	52	20	1	19	2	0	2	321	58	262
60–69	148	71	77	58	27	31	15	3	12	0	0	0	0	0	0	72	17	55
70–75	70	33	37	4	2	2	1	1	0	0	0	0	0	0	0	0	0	0
>75	0	0	0	4	2	2	1	0	1	0	0	0	0	0	0	0	0	0
BMI group																		
<18.5	34	10	24	9	0	9	18	0	18	6	0	6	7	0	7	16	0	16
18.5–24.9	425	190	235	219	47	172	213	24	189	128	0	128	104	19	85	548	63	485
25.0–29.9	323	193	130	54	20	34	52	19	33	21	4	17	11	2	9	222	58	164
≥30.0	217	109	108	25	11	14	12	1	11	5	0	5	2	1	1	87	16	71
Subjective health status																		
Poor	44	20	24	3	2	1	0	0	0	0	0	0	0	0	0	9	2	7
Fair	252	129	123	33	12	21	13	1	12	8	0	8	11	2	9	86	18	68
Good	453	217	236	107	23	84	84	14	70	42	1	41	29	6	23	358	52	306
Very good	216	115	101	125	29	96	159	24	135	86	3	83	61	12	49	335	50	285
Excellent	35	21	14	39	12	27	39	5	34	24	0	24	23	2	21	85	15	70

**Table 2:** Scores (%) achieved in each section and total score achieved for all study groups.

Subsample		Section 1 (Nutritional Recommendations)	Section 2 (Food Composition)	Section 3 (Food Choice)	Section 4 (Health Problems)	Total Score
Medical Doctors (*n* = 307)	M	75.73	78.05	86.32	88.49	81.29
	Md	77.78	77.78	84.62	90.48	81.82
	SD	10.45	8.04	10.54	7.33	5.65
	Min	38.89	50.00	53.85	57.14	60.23
	Max	100.00	97.22	100.00	100.00	93.18
Pharmacists (*n* = 295)	M	76.53	80.45	89.93	88.28	82.92
	Md	77.78	80.56	92.31	90.48	84.09
	SD	9.95	8.06	8.73	7.23	5.38
	Min	38.89	41.67	61.54	57.14	64.77
	Max	100.00	94.44	100.00	100.00	94.32
Dieticians (*n* = 160)	M	75.28	88.33	90.91	94.55	87.53
	Md	77.78	88.89	92.31	95.24	87.50
	SD	10.98	5.41	8.66	4.71	3.72
	Min	33.33	72.22	61.54	80.95	78.41
	Max	94.44	100.00	100.00	100.00	96.59
Nutritionists (*n* =124)	M	77.96	84.25	88.77	92.36	85.57
	Md	77.78	86.11	92.31	95.24	86.36
	SD	9.21	8.38	10.63	7.23	6.29
	Min	44.44	36.11	46.15	42.86	40.91
	Max	94.44	97.22	100.00	100.00	94.32
School teachers (*n* = 873)	M	70.71	70.66	83.19	78.99	74.51
	Md	72.22	72.22	84.62	80.95	75.00
	SD	13.04	11.49	12.99	12.75	9.51
	Min	11.11	8.33	0.00	0.00	9.09
	Max	94.44	97.22	100.00	100.00	94.32
General population (*n* = 1000)	M	61.12	57.42	65.38	66.04	61.41
	Md	66.67	58.33	69.23	71.43	63.64
	SD	16.97	15.10	21.21	16.87	14.11
	Min	0.00	0.00	7.69	4.76	2.27
	Max	94.44	88.89	100.00	95.24	88.64
Analysis of Covariance with age and sex as covariants		F (5;2750) = 108.934 *p* < 0.001	F (5;2750) = 361.568 *p* < 0.001	F (5;2750) = 197.777 *p* < 0.001	F (5;2750) = 307.705 *p* < 0.001	F (5;2750) = 392.103 *p* < 0.001
Bonferroni-corrected post-hoc analysis. *p* < 0.05		Nutritionists > dieticians = pharmacists = medical doctors > school teachers > general population	Dieticians > nutritionists > pharmacists > medical doctors > school teachers > general population	Dieticians = pharmacists = Nutritionists > medical doctors > school teachers > general population	Dieticians = nutritionists > pharmacists = medical doctors > school teachers > general population	Dieticians = nutritionists > pharmacists > medical doctors > school teachers > general population

Note: M = mean, Md = median, SD = standard deviation, Min = minimum, Max = maximum.

## Data Availability

All data presented in this manuscript and in the Supplemental Table S1.

## Supplementary Materials

The following supporting information can be downloaded at: https://www.mdpi.com/article/10.3390/nu14224729/s1, Table S1: Number of responses for each question of the German version of the General Nutrition Knowledge Questionnaire.
