# Supplementary material for: What Do People Know about Food, Nutrition and Health?—General Nutrition Knowledge in the Austrian Population
Source: Nutrients. 2022 Nov 9;14(22):4729. doi: 10.3390/nu14224729 (PMC9692599; doi:10.3390/nu14224729)
Supplement: Supplementary file 1 [file nutrients-14-04729-s001.zip › nutrients-2009805-Supplementary.pdf]

Supplementary Table S1: Number of responses for each question of the German version of the General Nutrition Knowledge Questionnaire (correct answers are marked in green).

Question	Options	Medical doctors	Pharma-cists	Dieti-cians	Nutritio-nists	School teachers	General popula-tion
		n	n	n	n	n	n
Total n							
Section 1: The first few items are about what advice you think experts are giving us.							
Do health experts recommend that people should be eating more. the same amount. or less of the following foods?							
fruit	not sure	7	2	6	0	15	17
	more	207	193	71	87	546	669
	same	82	76	70	32	270	260
	less	11	24	13	5	42	53
Food and drinks with added sugar	not sure	0	0	1	0	5	15
	more	2	0	1	0	5	19
	same	0	1	2	2	21	77
	less	305	294	156	122	842	889
Vegetables	not sure	0	0	1	0	4	11
	more	293	287	141	114	810	803
	same	14	8	18	10	59	150

	less	0	0	0	0	0	36
Fatty foods	not sure	3	4	3	0	10	18
	more	2	2	1	2	7	24
	same	27	40	17	15	84	164
	less	275	249	139	107	772	794
Processed red meat	not sure	5	1	1	2	40	80
	more	2	0	1	0	12	22
	same	14	18	2	2	94	198
	less	286	276	156	120	727	700
Wholegrains	not sure	1	1	1	0	16	25
	more	255	255	141	115	674	598
	same	50	38	18	6	171	318
	less	1	1	0	3	12	59
Salty foods	not sure	1	2	1	0	12	32
	more	1	0	0	1	2	26
	same	22	16	10	8	61	161
	less	283	277	149	115	798	781
Water	not sure	2	1	5	2	7	10
	more	236	229	110	73	743	824

	same	67	64	44	48	120	151
	less	2	1	1	1	3	15
How many servings of fruit and vegetables per day do experts advise people to eat as a minimum?							
servings of fruit and vegetables per day	2 servings	22	8	8	2	60	312
	3 servings	40	29	1	10	203	285
	4 servings	36	25	0	1	108	90
	5 or more servings	200	229	151	111	467	211
	not sure	7	4	0	0	32	102
Which of these types of fats do experts recommend that people should eat less of?							
unsaturated fats	not sure	5	2	0	1	94	266
	eat less	20	10	2	1	150	255
	not eat less	282	282	158	122	629	479
trans fats	not sure	19	6	0	0	180	353
	eat less	286	286	160	124	672	567
	not eat less	2	3	0	0	20	80
saturated fats	not sure	9	4	0	0	113	268
	eat less	280	274	159	121	607	531
	not eat less	18	17	1	3	153	201
Which type of dairy foods do experts say people should drink?							
	Full fat (e.g. full fat milk)	50	69	21	11	176	175

	Reduced fat (e.g. skimmed and semi-skimmed milk)	127	102	54	67	188	277
	Mixture of full fat and reduced fat	78	74	84	39	228	216
	Neither. dairy foods should be avoided	20	17	0	1	120	130
	Not sure	32	33	1	6	160	202
How many times per week do experts recommend that people eat oily fish (e.g. salmon and mackerel)?							
	1-2 times per week	272	257	160	122	791	815
	3-4 times per week	29	34	0	1	42	65
	Every day	0	0	0	1	0	8
	Not sure	6	4	0	0	39	112
Approximately how many alcoholic drinks is the maximum recommended per day (The exact number depends on the size and strength of the drink)?							
	1 drink each for men and women	164	159	49	32	640	652
	2 drinks each for men and women	1	6	2	0	19	71
	2 drinks for men and 1 drink for women	127	116	109	85	122	71
	3 drinks for men and 2 drinks for women	2	0	0	3	5	8
	Not sure	13	14	0	4	86	198
How many times per week do experts recommend that people eat breakfast?							
	3 times per week	8	2	0	0	6	8
	4 times per week	2	1	0	1	7	18
	Every day	284	273	157	118	829	932
	Not sure	13	19	3	5	30	42

If a person has two glasses of fruit juice in a day. how many of their daily fruit and vegetable servings would this count as?							
	none	103	107	29	23	188	184
	1 serving	35	33	20	26	109	214
	2 servings	60	66	73	57	241	268
	3 servings	60	38	30	11	169	132
	Not sure	49	51	8	7	166	202
According to the food guide pyramid[1] (a guideline showing the proportions of food types people should eat to have a balanced and healthy diet). how much of a person's diet should be made up of starchy foods?							
	Quarter	105	119	72	23	331	311
	Third	115	109	37	44	267	265
	Half	55	43	49	51	98	63
	Not sure	32	24	2	6	177	361
Section 2: Experts classify foods into groups. We are interested to see whether people are aware of food groups and the nutrients they contain.							
Do you think these foods and drinks are typically high or low in added sugar?							
Diet cola drinks	not sure	12	11	0	3	73	70
	High in added sugar	61	57	4	16	374	369
	Low in added sugar	234	227	156	105	426	561
Natural yoghurt	not sure	2	4	1	0	8	50
	High in added sugar	12	10	0	7	41	66
	Low in added sugar	293	281	159	117	824	884

[1] Wording was changed to Ernährungspyramide (food guide pyramid) since the "eatwell guide" is unknown in Austria

Icecream	not sure	1	1	0	0	2	20
	High in added sugar	306	294	160	123	867	952
	Low in added sugar	0	0	0	1	4	28
Tomato ketchup	not sure	0	2	0	0	1	32
	High in added sugar	302	293	155	123	864	920
	Low in added sugar	5	0	5	1	8	48
Melon	not sure	15	12	2	1	31	78
	High in added sugar	123	111	42	39	236	364
	Low in added sugar	169	172	116	84	606	558
Do you think these foods are typically high or low in salt?							
Breakfast cereals	not sure	35	33	3	9	148	235
	High in salt	80	81	27	30	237	259
	Low in salt	192	181	130	85	488	506
Frozen vegetables	not sure	17	12	4	4	43	134
	High in salt	61	38	4	12	192	219
	Low in salt	229	245	152	108	638	647
Bread	not sure	18	20	1	0	66	126
	High in salt	178	153	149	103	421	421
	Low in salt	111	122	10	21	386	453

Baked beans[2]	not sure	3	5	1	1	25	118
	High in salt	299	286	158	118	821	775
	Low in salt	5	4	1	5	27	107
Red meat	not sure	12	10	1	6	107	198
	High in salt	56	44	18	35	171	180
	Low in salt	239	241	141	83	595	622
Canned soup	not sure	0	1	0	1	11	96
	High in salt	307	294	160	123	857	842
	Low in salt	0	0	0	0	5	62
Do you think these foods are typically high or low in fibre?							
Oats	not sure	3	1	1	0	7	78
	High in fibre	303	292	159	122	850	846
	Low in fibre	1	2	0	2	16	76
Bananas	not sure	25	23	2	2	72	132
	High in fibre	135	140	83	86	473	586
	Low in fibre	147	132	75	36	328	282
White rice	not sure	6	6	1	1	35	122
	High in fibre	29	21	4	7	159	365

[2] Wording was changed to „Bohneneintopf in der Dose“ since baked beans are uncommon in Austria

	Low in fibre	272	268	155	116	679	513
Eggs	not sure	6	6	2	1	78	186
	High in fibre	1	4	0	2	58	238
	Low in fibre	300	285	158	121	737	576
Potatoes with skin	not sure	8	12	2	1	60	124
	High in fibre	248	251	140	115	730	693
	Low in fibre	51	32	18	8	83	183
Pasta	not sure	14	14	7	7	69	120
	High in fibre	29	25	26	21	267	497
	Low in fibre	264	256	127	96	537	383
Do you think these foods are a good source of protein?							
Poultry	not sure	2	2	0	1	35	81
	Good source of protein	294	288	160	118	769	814
	Not a good source of protein	11	5	0	5	68	105
Cheese	not sure	5	6	2	0	37	79
	Good source of protein	275	262	157	109	726	741
	Not a good source of protein	27	27	1	15	110	180
Fruit	not sure	2	5	0	1	42	119
	Good source of protein	5	6	0	1	23	101
	Not a good source of protein	300	284	160	122	807	780

Baked beans[3]	not sure	16	13	4	1	76	171
	Good source of protein	156	157	140	97	365	255
	Not a good source of protein	135	125	16	26	432	574
Butter	not sure	6	6	0	0	64	144
	Good source of protein	36	17	0	7	179	283
	Not a good source of protein	265	272	160	117	630	573
Nuts	not sure	16	19	3	3	65	110
	Good source of protein	225	216	118	86	626	749
	Not a good source of protein	66	60	39	35	181	141
Which of the following foods do experts count as starchy foods?							
Cheese	not sure	3	1	0	0	42	161
	Starchy food	19	9	0	1	68	240
	Not a starchy food	285	285	160	123	763	599
Pasta	not sure	0	0	0	0	3	47
	Starchy food	303	294	160	124	866	921
	Not a starchy food	4	1	0	0	4	32
Potatoes	not sure	1	2	0	0	5	70
	Starchy food	300	286	160	123	827	835

[3] Wording was changed to „Bohneneintopf in der Dose“ since baked beans are uncommon in Austria

	Not a starchy food	6	7	0	1	41	95
Nuts	not sure	14	8	1	2	61	133
	Starchy food	46	31	4	9	200	470
	Not a starchy food	247	256	155	113	612	397
Sweet potatoe[4]	not sure	16	12	2	3	97	179
	Starchy food	266	257	151	109	616	591
	Not a starchy food	25	26	7	12	160	230
Which is the main type of fat present in each of these foods?							
Olive oil	not sure	9	2	0	3	134	314
	Polyunsaturated fat	243	212	57	55	537	412
	Monounsaturated fat	51	80	103	62	138	165
	Saturated fat	4	1	0	4	61	100
	Cholesterol	0	0	0	0	3	9
Butter	not sure	14	7	0	1	140	290
	Polyunsaturated fat	7	4	1	0	42	105
	Monounsaturated fat	30	16	1	3	84	129
	Saturated fat	190	231	148	114	391	283
	Cholesterol	66	37	10	6	216	193

[4] Changed to sweet potatoe since plantains are uncommon in Austria

Sunflower oil	not sure	13	7	0	2	162	319
	Polyunsaturated fat	117	117	90	46	210	180
	Monounsaturated fat	145	157	69	70	358	295
	Saturated fat	32	14	1	6	124	164
	Cholesterol	0	0	0	0	19	42
Eggs	not sure	22	15	3	4	193	359
	Polyunsaturated fat	7	8	0	1	24	67
	Monounsaturated fat	34	22	3	4	90	116
	Saturated fat	36	25	62	30	99	107
	Cholesterol	208	225	92	85	467	351
Which of these foods has the most trans-fat?							
	Biscuits. cakes and pastries	271	284	159	120	680	486
	Fish	11	1	0	0	29	97
	Rapeseed oil	7	4	0	2	23	85
	Eggs	0	1	0	0	1	12
	Not sure	18	5	1	2	140	320
The amount of calcium in a glass of whole milk compared to a glass of skimmed milk is:							
	About the same	234	227	152	99	533	410
	Much higher	17	15	2	8	118	265
	Much lower	11	11	1	4	28	78

	Not sure	45	42	5	13	194	247
Which one of the following nutrients has the most calories for the same weight of food?							
	sugar	77	64	1	6	313	414
	starch	9	9	0	2	52	82
	dietary fiber	0	1	0	2	24	60
	fat	215	212	158	110	370	290
	not sure	6	9	1	4	113	154
Compared to minimally processed foods. processed foods are:							
	Higher in calories	266	252	150	111	649	212
	Higher in fibre	5	4	1	1	54	104
	Lower in salt	27	28	7	9	64	431
	Not sure	9	11	2	3	105	253
Section 3: The next few items are about choosing foods							
If a person wanted to buy a yogurt at the supermarket. which would have the least sugar/sweetener?							
	0% fat cherry yogurt	1	1	1	1	12	61
	Natural yogurt	306	294	158	122	846	837
	Creamy fruit yogurt	0	0	0	1	2	23
	Not sure	0	0	1	0	13	79
If a person wanted soup in a restaurant or cafe. which one would be the lowest fat option?							
	Mushroom risotto soup (field mushrooms. porcini mushrooms. arborio rice. butter. cream. parsley and cracked black pepper)	5	1	1	0	21	70

	Carrot butternut and spice soup (carrot . butternut squash. sweet potato. cumin. red chillies. coriander seeds and lemon)	290	289	158	122	801	665
	Cream of chicken soup (British chicken[5]. onions. carrots. celery. ≤ potatoes. garlic. sage. wheat flour. double cream)	10	4	0	1	30	139
	Not sure	2	1	1	1	21	126
Which would be the healthiest and most balanced choice for a main meal in a restaurant?							
	Roast turkey. mashed potatoes and vegetables	291	276	157	117	794	729
	Beef. Yorkshire pudding and roast potatoes [6]	12	16	2	5	55	151
	Fish and chips served with peas and tartar sauce	2	2	0	0	3	54
	Not sure	2	1	1	2	21	66
Which would be the healthiest and most balanced sandwich lunch?							
	Ham sandwich + fruit + blueberry muffin + fruit juice	7	3	1	0	20	67
	Tuna salad sandwich + fruit + low fat yogurt + water	293	290	159	124	834	840
	Egg salad sandwich + crisps + low fat yogurt + water	1	0	0	0	2	29
	Not sure	6	2	0	0	15	64
Which of these foods would be the healthiest choice for a pudding?							

[5] „British chicken“ was changed to Hühnerfleisch aus Österreich
[6] Wording was changed to „Tafelspitz mit Semmelkren und Bratkartoffeln“ since Yorkshire Pudding is uncommon in Austria

	Berry sorbet	236	214	131	104	605	502
	Apple and blackberry pie	7	13	6	1	35	62
	Lemon cheesecake	4	2	3	2	19	42
	Carrot cake with cream cheese topping	43	40	12	8	140	263
	Not sure	17	26	8	9	74	131
Which of these combinations of vegetables in a salad would give the greatest variety of vitamins and antioxidants?							
	Lettuce. green peppers and cabbage	42	23	18	6	127	161
	Broccoli. carrot and tomatoes	190	226	120	88	500	445
	Red peppers. tomatoes and lettuce	31	35	6	17	98	205
	Not sure	44	11	16	13	148	189
If a person wanted to reduce the amount of fat in their diet. but didn't want to give up chips. which of the following foods would be the best choice?							
	Thick cut chips	231	231	126	86	561	366
	Thin cut chips	21	20	13	20	65	202
	Crinkle cut chips	10	3	4	4	34	87
	Not sure	45	41	17	14	213	345
One healthy way to add flavour to food without adding extra fat or salt is							
	Coconut milk	1	0	0	0	12	72
	Herbs	300	292	159	123	817	753
	Soya sauce	3	2	1	1	31	88

	Not sure	3	1	0	0	13	87
Which of the following cooking methods requires fat to be added?							
	Grilling	2	5	3	0	10	44
	Steaming	1	0	0	2	4	55
	Baking	60	39	33	7	138	141
	Sautéing	239	249	119	113	706	760
	Not sure	5	2	5	2	15	0
Traffic lights are often used on nutrition labelling. what would amber mean for the fat content of a food?							
	Low fat	4	0	1	3	8	36
	Medium fat	262	273	157	116	738	703
	High in fat	10	1	0	1	10	44
	Not sure	31	21	2	4	117	217
“Light” foods (or Diet foods) are always good options because they are low in calories.							
	Agree	15	8	6	14	36	138
	Disagree	281	282	154	105	796	706
	Not sure	11	5	0	5	41	156
Looking at products 1 and 2. which one has the most calories (kcal) per 100 grams							
	Product 1	239	253	134	95	660	556
	Product 2	51	34	23	24	158	308

	Both have the same quantity	7	4	3	5	17	51
	Not sure	10	4	0	0	37	85
Looking at product 1. what are the sources of sugar in the ingredient list?							
	Sugar and malt syrup	13	13	1	7	39	118
	Sugar. fructose and lecithin	2	0	0	0	21	89
	Sugar. fructose and malt syrup	287	280	159	116	783	638
	Not sure	5	2	0	1	30	155
Section 4: This section is about health problems or diseases related to diet and weight management							
Which of these diseases is related to a low intake of fibre?							
	Bowel disorders	297	294	156	122	777	664
	Anaemia	1	0	0	1	25	68
	Tooth decay	6	1	4	0	2	37
	Not sure	3	0	0	1	69	231
Which of these diseases is related to how much sugar people eat?							
	High blood pressure	18	7	2	2	103	278
	Tooth decay	286	285	157	120	733	616
	Anaemia	1	0	0	0	3	17
	Not sure	2	3	1	2	34	89
Which of these diseases is related to how much salt (or sodium) people eat?							
	Hypothyroidism	3	3	2	1	132	258

	Diabetes	0	0	0	0	4	56
	High blood pressure	303	291	158	122	615	489
	Not sure	1	1	0	1	122	197
Which of these options do experts recommend to reduce the chances of getting cancer?							
	drinking alcohol regularly	0	0	0	0	3	21
	eating less red meat	224	194	154	121	375	448
	avoding additives in food	74	92	4	2	416	373
	not sure	9	9	2	1	79	158
Which of these options do experts recommend to prevent heart disease?							
	Taking nutritional supplements	1	1	0	1	3	37
	Eating less oily fish	11	3	0	1	29	107
	Eating less trans-fats	293	290	160	120	787	693
	Not sure	2	1	0	2	54	163
Which of these options do experts recommend to prevent diabetes?							
	Eating less refined foods	302	294	159	121	788	722
	Drinking more fruit juice	2	1	1	1	14	55
	Eating more processed meat	1	0	0	1	6	28
	Not sure	2	0	0	1	65	195
Which one of these foods is more likely to raise people's blood cholesterol?							

	Eggs	47	39	5	16	123	217
	Vegetable oils	1	2	1	1	12	59
	Animal fat	254	250	154	105	681	593
	Not sure	5	4	0	2	57	131
Which one of these foods is classified as having a high Glycaemic Index (Glycaemic Index is a measure of the impact of a food on blood sugar levels. thus a high Glycaemic Index means a greater rise in blood sugar after eating)?							
	Wholegrain cereals	12	8	6	3	56	82
	white bread	287	281	153	119	706	603
	Fruit and vegetables	5	4	1	1	21	64
	Not sure	3	2	0	1	90	251
To maintain a healthy weight people should cut fat out completely							
	agree	3	2	0	0	13	91
	Disagree	302	292	160	124	852	828
	Not sure	2	1	0	0	8	81
To maintain a healthy weight people should eat a high protein diet.							
	agree	193	198	73	56	571	631
	Disagree	101	79	83	65	214	207
	Not sure	13	18	4	3	88	162
Eating bread always causes weight gain.							

	agree	11	8	0	1	53	146
	Disagree	289	284	160	123	791	759
	Not sure	7	3	0	0	29	95
Fibre can decrease he chances of gaining weight.							
	agree	258	262	151	114	612	517
	Disagree	24	20	9	7	123	210
	Not sure	25	13	0	3	138	273
Which of these options can help people to maintain a healthy weight?							
Not eating while watching TV	not sure	2	2	0	0	13	47
	Yes	275	272	143	109	778	806
	No	28	21	17	15	77	147
Reading food labels	not sure	7	6	0	2	12	49
	Yes	286	282	152	113	829	860
	No	10	7	8	9	29	91
Taking nutritional supplements	not sure	10	18	0	4	55	121
	Yes	8	41	0	5	45	167
	No	285	234	160	115	766	712
Monitoring their eating	not sure	4	1	1	1	6	37
	Yes	300	290	156	117	856	927

	No	2	4	3	6	7	36
Monitoring their weight	not sure	8	11	4	1	19	36
	Yes	272	265	141	115	781	900
	No	25	19	15	8	68	64
Grazing throughout the day	not sure	1	0	0	0	4	32
	Yes	1	1	1	0	3	29
	No	301	292	159	124	859	939
If someone has a Body Mass Index (BMI) of 23kg/m^2. what would their weight status be?							
	underweight	6	7	0	0	24	60
	normal weight	277	248	159	123	565	469
	overweight	17	31	1	1	71	115
	obese	0	1	0	0	2	356
	not sure	7	8	0	0	211	0
If someone has a Body Mass Index (BMI) of 31kg/m^2. what would their weight status be?							
	underweight	1	0	0	0	4	14
	normal weight	2	2	0	1	17	57
	overweight	82	80	17	32	310	614
	obese	220	205	143	91	343	315
	not sure	2	8	0	0	199	0

Which of these body shapes increases the risk of cardiovascular disease (Cardiovascular disease is a general term that describes a disease of the heart of blood vessels. for example. angina. heart attack. heart failure. congenital heart disease and stroke)?							
	Apple shape	293	285	159	122	773	801
	Pear shape	11	9	1	1	25	85
	Not sure	3	1	0	1	75	114
